# Preserving cultural heritage: A community-centric approach to safeguarding the Khulubvi Traditional Temple Malawi

**DOI:** 10.1016/j.heliyon.2024.e37610

**Published:** 2024-09-10

**Authors:** Lazarus Obed Livingstone Banda, Chigonjetso Victoria Banda, Jane Thokozani Banda, Tapiwa Singini

**Affiliations:** a Beijing Institute of Technology, School of Social Science and Humanities, LiangXiang Campus, Beijing, China; b University of Malawi, School of Political Science and Administrative Studies, Malawi; c Ministry of Education, Directorate of Higher Education, Lilongwe, Malawi; d East China Normal University, School of Social Development, Department of Anthropology, Shanghai, China

**Keywords:** Community-based participatory research, Malawi cultural heritage, Heritage management, Intergenerational knowledge transfer, Cultural heritage preservation, Community engagement strategies

## Abstract

The study investigates cultural heritage conservation through community-based participatory research, focusing on preserving the Khulubvi Traditional Temple. It addresses challenges from religious, societal, and economic changes and the importance of integrating heritage into education. It emphasizes technology's role in maintaining sacred narratives. Qualitative methods, such as interviews and thematic analysis, reveal community efforts and modern challenges. The study concludes with a call to embed heritage in formal education and highlights the community's crucial role in cultural legacy, contributing to the discourse on heritage preservation.

## 1 Introduction

Cultural heritage (CH), a continuum of traditions and practices passed down through generations, encapsulates the essence of a society's collective memory. It refers to the legacy of physical artifacts and intangible attributes of a group or society that are inherited from past generations, maintained in the present, and bestowed for the benefit of future generations [1,2]. Cultural heritage includes tangible forms, intangible forms, and natural heritage [3]. Tangible CH includes buildings and historical places, monuments, artifacts, etc., which are considered worthy of preservation for the future. These are physical artifacts that were created, maintained, or leftover by the cultures of the past. Examples include museums, architectural monuments, archaeological sites, and historic cities. Intangible CH includes practices, representations, expressions, knowledge, and skills—as well as the associated instruments, objects, artifacts, and cultural spaces—that communities, groups, and, in some cases, individuals recognize as part of their CH. This aspect includes traditions or living expressions inherited from ancestors and passed on to descendants, such as oral traditions, performing arts, social practices, rituals, festive events, knowledge and practices concerning nature and the universe, or the knowledge and skills to produce traditional crafts.

Culture is a dynamic and multifaceted construct, reflecting a group or society's shared beliefs, values, customs, traditions, and social norms [[4], [5], [6]]. It constitutes a learned, negotiated system impacting societal thought processes and interactions [7]. It evolves through interactions and exchanges with other cultures, forming a continuous process of negotiation and expression [8]. This ongoing adaptation and transmission of cultural elements underscore their pivotal role in individual and collective identity formation within specific societal contexts [9]. Before advancing further in this discourse, it is imperative to delineate and distinguish between two pivotal terms frequently employed within the ambit of CH management: 'CH preservation' and 'CH conservation.

On the one hand, CH conservation is more focused on the active treatment, a more physical or material aspect of heritage management, and repair of tangible cultural artifacts or monuments to restore them to a known or assumed state, such as repairing the Mbona Shrine hut and the house of his wife and the maid, or reforestation, often to enhance legibility or usability while retaining its heritage value. It comprises an array of actions and measures to sustain its existence, reinforcing the enduring preservation of its inherent messages and values [10]. Conservation includes both the preservation of the fabric of the object huts or the forest and the stabilization from further deterioration.

On the other hand, CH preservation generally refers to the protection, maintenance, and recovery of CH without altering it to ensure its survival for future generations. Preservation often aims to maintain the current state of the heritage site or artifact and can include measures to prevent change or deterioration. This involves avoiding poaching, encroachment, and logging in the forest environment and around the artifacts. It also involves protecting intangible cultural practices by cleaning, repairing, or restoring them, researching and analyzing the artifacts to understand their original context and condition, and recording, documenting, or supporting the continued practice of these traditions in their community context.

The word community has been contested and defined in various ways in conventional literature. However, in the current study, we restrict the term “community” to the Mang'anja and non-Mang'anja living around the Khulubvi. Much as the Mbona traditions and customs concern others, even those in the diaspora, those in the urban sector or away are beyond this study's interest. This study concerns the preservation and conservation of the Khulubvi rain shrine and the associated tangible and intangible artifacts practices.

Mbona, a revered figure in Malawian mythology known for his prowess as a rainmaker and guardian of nature [[11], [12], [13], [14], [15]], attributed with supernatural powers [12,14,16,17]), including the capacity to summon rain, create wells in sandy terrains, transform barren lands into lush forests, and even metamorphose adversaries into various animals, such as guinea pigs, for evasion, imparts several vital teachings that are integral to the cultural fabric of the Mang'anja and other southern Malawian ethnic groups. The following are among the central issues the study focuses on in an attempt to unravel the preservation and conservation of His shrine:

Central to Mbona's teachings is the principle of harmony with nature, emphasizing the importance of living in balance with the environment to ensure agricultural fertility and sustainability. This ecological stewardship is intertwined with moral and ethical living, where Mbona underscores the values of honesty, justice, and fairness as foundational to social cohesion and community well-being. Additionally, Mbona's legacy includes a strong focus on spiritual practices, advocating for the observance of rituals that bridge the physical and spiritual realms, thereby maintaining cosmic balance and protecting the community. 10.13039/100014337Furthermore, Mbona champions community unity and support, teaching the importance of mutual assistance and collective strength.

The rest of this study is structured into the following subsections: Literature review, theoretical framework, geographical setting, materials and methods, results, discussion, and conclusion.

## 2 Literature review

### 2.1 Veneration in the Mbona cult at Khulubvi

In African traditional religions, ancestor veneration is a foundational element of CH, profoundly expressing the ongoing relationship between the living and their forebears [18]. This practice influences moral behavior and societal harmony by imposing expectations of moral conduct. It entails a respectful engagement with the spirits of deceased ancestors, who are believed to continue exerting influence over the lives of their descendants [18,19]. This Veneration underscores the belief that living morally and exemplarily per community values can lead to a posthumous status as an ancestor, providing spiritual guardianship and moral guidance to living relatives. The practice supports social cohesion and instills a framework where personal and collective well-being is interlinked with ethical living, illustrating the profound integration of spiritual beliefs with practical moral outcomes [19].

This Veneration facilitates a dynamic and continuous interaction between the tangible and spiritual realms through various acts of remembrance and respect, such as rituals and ceremonies. This interaction sharply contrasts with Western interpretations, which frame such spiritual engagements within a dichotomous paradigm of religious worship or demonic activity [18].

The shrines and sacred sites in Malawi date to 1500 AD [11,16], wherein ancestral communities utilized these locations to offer sacrifices to their deities during periods of famine or other calamities, as documented in the historical account of Mphambe. Mphambe is a name commonly associated with God's manifestations of power in nature, mainly through lightning, thunder, storms, and earthquakes [20,21]. This term, derived from the verb "-pambana," meaning "to exceed" or "excel," emphasizes the surpassing greatness and awe-inspiring power attributed to Mphambe [21]. In the cultural and religious practices of the Chewa people, Mphambe represents both a revered and feared divine presence, deeply rooted in their understanding of the natural world and its phenomena [20,21]. The Chewa people believe in a Supreme God, known by various names, including Mulungu, Namalenga, Leza, Chisumphi, and Mphambe. This Supreme God maintains an active interest in human affairs through ancestral spirits, who act as intermediaries between the divine and human realms [22]. This belief system emphasizes the interconnectedness of the natural world, the divine, and human experiences, particularly in the context of ecological and environmental phenomena [22]. Similar to Bimbi among the Chewa of the south [22] and Chisumphi among the Chewa [23], which venerate nature spirits, Khulubvi Sacred Shrine holds profound cultural and religious significance for the Mang'anja Tribe, as it is dedicated to the Veneration of divinized human of Mbona [24],

The Mbona cult is characterized by a ritual cycle intricately interwoven with the political and social fabric of the region. Central to this cult is the shrine, which is erected over the location where Mbona's head is purported to have been buried [originally bare, without any tree], and his blood turned into a stream, marking these sites sacred [25]. According to local legend, the bare ground was transformed overnight into a dense forest known as Khulubvi. This transformation is believed to be a manifestation of Mbona's spiritual potency, with the forest—reputedly sprouted from the hair of Mbona's buried head—serving as a tangible manifestation of his enduring influence and his pivotal role as both a cultural and a spiritual icon.

Like the Kuomboka, African myths are not short of diverse versions [12,26]. Varied as their versions are [12], the myths and traditions, falling priestly, chiefly, and royal—each maintaining distinct versions of the Mbona legend according to their societal roles [25], serve as both spiritual guidance and historical recounts, shaping the political and cultural identity of the Mang'anja people [25]. Each 'stream' is associated with different levels of political centralization and linked to the north-south movement in the Shire Valley [23].

### 2.2 Who benefits from conserving and preserving cultural heritage?

Firstly, it is paramount to indicate that although CH should be the pride of the host community, not all conservation or preservation measures benefit the indigenous populations [27,28]. Besides, not all aspects of heritage should be preserved. Threats to CH worldwide include war, development, climate change, tourism, natural disasters, and looting.

Secondly, some scholars contend that CH necessitates preservation due to its inherent vulnerability. They use terms such as "[cultural] heritage at risk" [29,30], while others critique this premise as a pretext for concealed agendas, critically examining the preservation methodologies promoted to mitigate perceived risks [30]. There is a need to scrutinize the political and social dimensions of heritage preservation, noting that what is considered "at risk" often reflects specific interests and power dynamics rather than an objective assessment of threats [30]. Rico acknowledges how the rhetoric of risk can obscure the real issues at stake and may lead to the exclusion of critical cultural narratives and practices.

Subsequently, we must advocate for preservation practices that reflect and respond to the complexities of CH and its various stakeholders [30]. Therefore, care must be taken when choosing the aspects and the measures.

Nonetheless, CH sites are central to performing numerous traditional expressions, including initiation ceremonies for young boys. As such, the youths benefit from essential moral and social education, thus facilitating their seamless transition into adulthood and equipping them with the requisite knowledge and behaviors expected in mature societal roles [31]. The entire community indirectly benefits from the continuance and reinforcement of its CH and social structure [31]. Nonetheless, not all ages, role players, or members of the community value different CH the same way [32]. For example, while the elderly women at Khulubvi may be proud of dancing with their breasts wholly exposed in the presence of men, some of their children may not stand the “shame,” indicating a moral and CH dissonance [32].

Besides, Khulubvi sites are focal points for cultural exchange and traditional practices, attracting diverse individuals from various cultural backgrounds. Such practices underline the cultural richness and communal significance of the Khulubvi Forest, advocating for its preservation not only as a biological haven but also as a living repository of CH [11].

Furthermore, the traditional prohibitions against encroachment and poaching contribute significantly to ecological stability [33]. These cultural taboos support the reduction of greenhouse gas emissions, enhancing the water cycle and preserving local fauna, which is culturally revered as the progeny of Mbona. Such traditions uphold cosmic stability and intertwine cultural practices with environmental conservation, demonstrating a symbiotic relationship between CH and ecological sustainability.

As an area of particular interest, tourists and scholars in CH are a source of visas and related costs. However, it is unclear how the Mang'anja community benefits from this compared to the rest of Malawi, a case similar to Chongoni in Malawi [34] and Kuomboka among the Lozi of Zambia [26].

### 2.3 Challenges associated with cultural heritage preservation in general

Despite its intrinsic value, the preservation of CH faces multifaceted challenges, especially in the wake of globalization, migration, and rapid industrialization [26,35]. The scope of challenges encountered encompasses issues that demand sophisticated approaches to cartographic documentation, continuous monitoring, and facilitation of access. Furthermore, the endeavor of CH preservation is complicated by the imperative for a compelling presentation that respects and conveys the value of the heritage and the complexities associated with determining and managing ownership rights [36]. For instance, since the custodians believe that the Khulubvi Forest is not a natural thicket but from the hair of their ancestors and the associated river as the blood of their martyr, other communities, organizations, or government arms may be viewed as intruders in managing the site.

Several pivotal challenges are evident, exemplified by cases such as Vimbuza, but they are broadly applicable across similar cultural phenomena. A primary concern is preserving authenticity and integrity as these practices transition into the public and international domains. The essence of rituals, deeply rooted in specific cultural and spiritual systems, risks dilution or misinterpretation when showcased merely as cultural performances, leading to a loss of the original contextual meanings and functions that define their roles within native communities [37]. Besides, social and economic development makes some social services that were once rare and expensive more affordable and accessible. For example, youths who believe in healing through spirits nowadays prefer hospitals, corroborating literature on the dissonance in heritage [32]. This is well explained from modernization perspectives [[38], [39], [40], [41]]. Similar challenges have been traced among the youths of Namibia [35] who mix local values with foreign practices. This is particularly dangerous for the Mbona cult as contravening the teachings infuriates Mbona, who retaliates wrathfully. Therefore, exposure to foreign cultures must be treated with caution, maintaining a healthy dynamic equilibrium [16,17,31,35,42,43].

There is a tension between preservation and the natural evolution of cultural practices. Overly strict regulations might prevent cultural practices from evolving and adapting to contemporary contexts, potentially leading to cultural stagnation [44].

Besides, spiritual practices gain recognition on platforms like UNESCO's lists. They are subject to interpretations and representations that may not align with indigenous understandings. This global exposure, while beneficial in raising awareness, can also lead to significant cultural misunderstandings, altering the perception of these practices among both local adherents and the global audience. Such misunderstandings can provoke conflicts of cultural identity and heritage authenticity, challenging the indigenous narrative and self-perception [37]. There is often a significant disconnect between global policies and local realities. The massive international scale of organizations and their metacultural policies can sometimes be at odds with local, grassroots practices and perceptions. This can lead to policies that are not effectively implemented or accepted locally because they do not resonate with or even acknowledge local needs, contexts, and practices [37].

Moreover, the commercialization of intangible spiritual heritage poses significant challenges. As rituals and practices become commodified, their sacred elements may be overshadowed by economic motivations, leading to a transformation prioritizing entertainment value over spiritual and therapeutic efficacy. Commodification affects the practice's spiritual depth, cultural significance, and continuity, potentially diminishing its role as a cohesive element within the community [26,45,46]. Besides, the influx of tourists may lead to an increase, making goods and services unaffordable to the locals [26]. The influx may also increase pressure on the limited resources in the community, leading to logistical challenges [26,35], much as it may also lead to positive infrastructure development [35]. Unless such development is handled to ensure sustainable ecotourism, it may lead to the loss of some CH.

These spiritual practices are often integral to communities' cultural identity and cohesion. International recognition and the subsequent alteration of these practices can impact community identity, sometimes strengthening it through pride in global recognition but often challenging it by changing the practice's role and significance within its original context [47].

Cognizant of human rights and freedoms, preserving some practices is challenging. Human rights must be understood as universally valid, transcending specific contextual limitations. Their legitimacy stems inherently from the fundamental nature of human beings rather than being contingent upon different populations' varying socio-economic, cultural, and political conditions. Thus, the essence and enforcement of human rights are not defined or constrained by the particular circumstances of a society. So, preservation efforts must contribute to promoting cultural diversity and tolerance, illustrating the importance of nurturing distinct cultural expressions in a rapidly globalizing world [36].

Managing the balance between local values and global recognition remains a delicate task. Ensuring that spiritual practices are respected and preserved according to local terms while engaging with global initiatives like UNESCO requires nuanced strategies. These strategies must prioritize the voices and preferences of the indigenous communities to maintain the practice's integrity and relevance, supporting rather than undermining their traditional roles [47]. Besides, the benefits and support provided by heritage policies can be unevenly distributed, leading to inequities within communities. Specific individuals or institutions might gain more from these policies than others, which can exacerbate existing social divisions or create new ones [44].

These challenges underscore the complexity of preserving intangible CH, especially those with deep spiritual significance. They highlight the need for culturally sensitive approaches that respect the values and expectations of the communities from which these practices originate [47].

### 2.4 Challenges within the Mbona Cult

Malawians have increasingly been intermarrying across cultures. Besides, there are some international visitors. As seen in the case of Namibia, such social activities impact CH, making the youth like foreign music, fashion, and media more than adhering to local values, admiring traditional and ritual-related dressing, and other spiritual values [35]. Literature records the decline of rain shrines in Malawi, including that of the Chewas around Bunda and the Mbona of the Mang'anja, due to opposing forces or available persevering alternatives [43].

There are intricate dynamics between traditional authority and popular religious movements [47]within the Mbona cult: Traditional authority is vested in local chiefs responsible for maintaining the shrine's physical integrity and orchestrating community rituals. These chiefs symbolize the entrenched political and social structures, wielding significant influence over communal assemblies and allocating communal resources.

In stark contrast, the spirit medium (Mulozi) emerges as a pivotal figure, characterized by an absence of formal appointment and the capacity to garner substantial popular support. This individual acts as an intermediary between Mbona's spirit and the community, especially in times of crisis, such as drought or disease outbreaks. The legitimacy of the spirit medium stems not from traditional hierarchical structures but from an ability to mobilize community sentiment and articulate collective desires and grievances [17].

The interaction between the chiefs and the spirit medium is fraught with tension and conflict. As embodiments of conventional authority, the chiefs may perceive the spirit medium as a destabilizing force that challenges their control, mainly when it influences public opinion and initiates community action that contravenes the chiefs' directives. Conversely, the spirit medium often positions itself as a vocal critic of the chiefs, especially when their actions are considered self-serving or detrimental to the collective well-being [17].

This dialectical relationship highlights a broader theme prevalent in many societies, where formal, structured leadership coexists and sometimes clashes with more spontaneous, charismatic forms of authority. In the Mbona cult, the spirit medium serves not only as a mechanism for social expression and crisis management but also as a check on the power exerted by traditional chiefs.

Environmental factors play a critical role in shaping the trajectory of cultural evolution. Understanding this dynamic offers insights into the mechanisms by which cultures adapt or resist change, highlighting the importance of environmental contexts in preserving or transforming cultural identities. This dynamic equilibrium [a synthesis of historical continuity and modern realities] perspective enriches our comprehension of cultural dynamics, providing a framework to anticipate and understand cultural responses to environmental shifts [16,17,31,42,43].

Lastly, Much as tangible heritage has remained intact, their myths have undergone diverse interpretations, influenced by evolving political and social landscapes and their utilization in reinforcing historical and cultural identities. His discourse suggests that without deliberate preservation efforts, these narratives will likely diverge significantly over time, reflecting shifts in cultural and historical contexts.

### 2.5 Relevance of the Khulubvi Sacred Rain Shrine warranting preservation

1. Mbona narratives ought to be considered intricate syntheses of cultural memory, spiritual significance, and historical events deeply entrenched within cultural myths, which perform dual roles of educating and legitimizing extant social structures and interpretations of history [12,25,31,48].
2. Importantly, Khulubvi Rain Shrine is integral to the ceremonial enthronement of the Mang'anja's Paramount Chief [16,17], highlighting its role in the governance and cultural leadership within the community, in a similar way that the Bimbi cult is among those venerating the same [22].
3. In Malawi, issues about witchcraft are controversial. The cult serves as a symbolic authority in dispute resolutions that involve elements of witchcraft and spirit possession; hence, it is vital to influence the policy framework for formal and informal legal proceedings to interpret and address claims of supernatural occurrences [15].
4. Besides, the cult's influence helps to legitimize traditional practices within the broader legal framework, ensuring that cultural identity and heritage are preserved within modern legal processes [15]. This integration helps maintain social harmony by respecting the community's spiritual beliefs while aligning them with national legal frameworks.
5. This shrine is a pivotal spiritual hub, primarily linked with essential rainmaking rituals. Such rituals are indispensable in an agrarian society that heavily depends on seasonal rainfall for agriculture. The erosion of heritage thus represents a severance of a population's ties to its historical dimensions, jeopardizing the perpetuation of said heritage and potentially leading to deviations from traditional conceptions of heritage [49].
6. It also stands as a bastion of historical and cultural continuity for the Mang'anja people [25], holding significant importance for scientific study and biodiversity conservation, primarily due to its unique status where encroachment and poaching are culturally taboo. This forest is distinguished by its singular historical relevance in the broader Southern African region. As a vital resource, it offers extensive opportunities for interdisciplinary research across both social and natural sciences [46].
7. The shrine represents a profound cultural and spiritual asset, particularly revered by the Mang'anja community in the southern region of the nation [12,16,17,25], serving as a unique window into the historical and political evolution of southern Malawi, showing how local communities have responded to and been shaped by changes over centuries [16]. In the whole of Southeast Africa [46]. Neglect of such heritage critically impacts the resilience of cultural identities and the welfare of vulnerable communities [50]. Therefore, this preservation is crucial not only for academic study but also for the cultural identity of the local population [17,25].
8. The redirection of community interests, whether inadvertent or deliberate, or under the influence of human rights, results in marginalizing the past and its historical signifiers from prominent discourse, as these elements are either omitted, altered, or rewritten [36,51]. As such, maintaining the shrine ensures that future generations can engage with and learn from this reservoir of traditional knowledge, thereby fostering a deeper understanding of their ancestral heritage.
9. Additionally, while the primary rationale for the shrine's preservation should focus on cultural integrity, there exists potential for developing respectful and sustainable cultural tourism. Such an initiative could benefit the local community economically while educating visitors about Mang'anja's rich CH. However, commercialization must be done with much care.

In essence, the conservation of Khulubvi Rain Shrine is not merely an act of preserving a physical location but a critical endeavor to maintain the cultural, historical, and spiritual fabric of the Mang'anja community. It is a fundamental step towards ensuring that the heritage and identity of the Mang'anja, as well as the broader cultural landscape of Malawi, are celebrated, understood, and transmitted to succeeding generations. This intertwined approach to the shrine's significance and the imperatives for its preservation underscores its pivotal role in cultural identity and educational outreach.

Recognizing its cultural and historical importance, the Khulubvi Traditional Temple was included in the tentative list of world heritage sites in 2011, and its formal nomination is underway. Traditional Temple in Malawi, a repository of ancient beliefs and practices. Historically, communities reverently worshipped natural phenomena, seeking solace during calamities. However, the advent of colonialism and foreign religions such as Christianity led to a shift [32], eroding traditional custodial rights and disrupting age-old practices. While remnants of sacred sites like Khulubvi, Bimbi, and Kyala in Mbande Hills (a deity among the Ngondes of Karonga, a god of rain and harvest) remain neglected, their sanctity is fading under modernization and economic necessity pressures.

Considering these challenges, this study explores the pivotal role of community participation in managing the Khulubvi Traditional Temple. By delving into residents' perceptions and active involvement, the study aims to identify sustainable strategies for CH preservation. The research objectives are threefold.

1. To assess community engagement
2. To examine stakeholder operations.

This research bridges an essential gap by focusing on the involvement of local communities in preserving and conserving CH, specifically in developing countries like Malawi. By exploring the nuanced dynamics between heritage preservation, community engagement, and external interventions, this study contributes meaningful insights to the global discourse on CH preservation. Through this exploration, we aim to shed light on practical strategies that empower communities to safeguard their heritage for future generations, ensuring the continuity of their cultural legacy.

### 2.6 Research questions

1. What specific CH preservation activities are undertaken by residents to ensure the sustainability and promotion of the Khulubvi Traditional Temple in Malawi?
2. How do various stakeholders, including parties, NGOs, religious institutions, and village representatives (VRs), operate to safeguard CH, mainly focusing on the Khulubvi Traditional Temple?
3. What are the distinct challenges encountered in preserving the Khulubvi Traditional Temple?
4. What pragmatic solutions can be proposed to enhance its preservation and conservation, considering both tangible and intangible aspects of CH?

## 3 Theoretical framework

Based on the research objectives and questions, this qualitative study was situated against the Community-Based Participatory Research (CBPR) theoretical framework. CBPR is a collaborative research approach that involves the active participation of community members, organizational representatives, and researchers in the research process [52]. It emphasizes the importance of involving the community in identifying research questions, data collection, analysis, and decision-making. CBPR aligns well with the objectives and questions of the study in the following ways: Firstly, it emphasizes the active involvement of community members, which aligns with the study's aim to examine and evaluate community participation in preserving and conserving the Khulubvi Traditional Temple. Secondly, CBPR allows for in-depth exploration of residents' perceptions and participation, addressing the specific objective of identifying residents' perceptions and participation in preserving and conserving CH. Thirdly, the framework enables the identification and understanding of local community involvement processes and strategies concerning protecting CH, aligning with another specific study objective.

Furthermore, CBPR facilitates collaborative problem-solving [53], allowing the exploration of challenges faced in preserving and conserving CH and generating possible solutions, as outlined in the research questions. In addition, CBPR involves various stakeholders, such as traditional leaders, NGOs, and community organizations [54]. This aligns with the research question about how different parties safeguard CH. Besides, the theory promotes sustainability: It emphasizes sustainable solutions by involving the community in decision-making and implementation, ensuring that the preservation and conserving efforts are long-lasting and effective.

For these reasons, incorporating the CBPR framework allowed the researchers to actively engage with the community, gain valuable insights from community members, and collaboratively work towards solutions for preserving and conserving the Khulubvi Traditional Temple, making it a suitable choice for this qualitative study.

## 4 Geographical setting of Khulubvi Sacred Rain Shrine

**Fig. 1:**
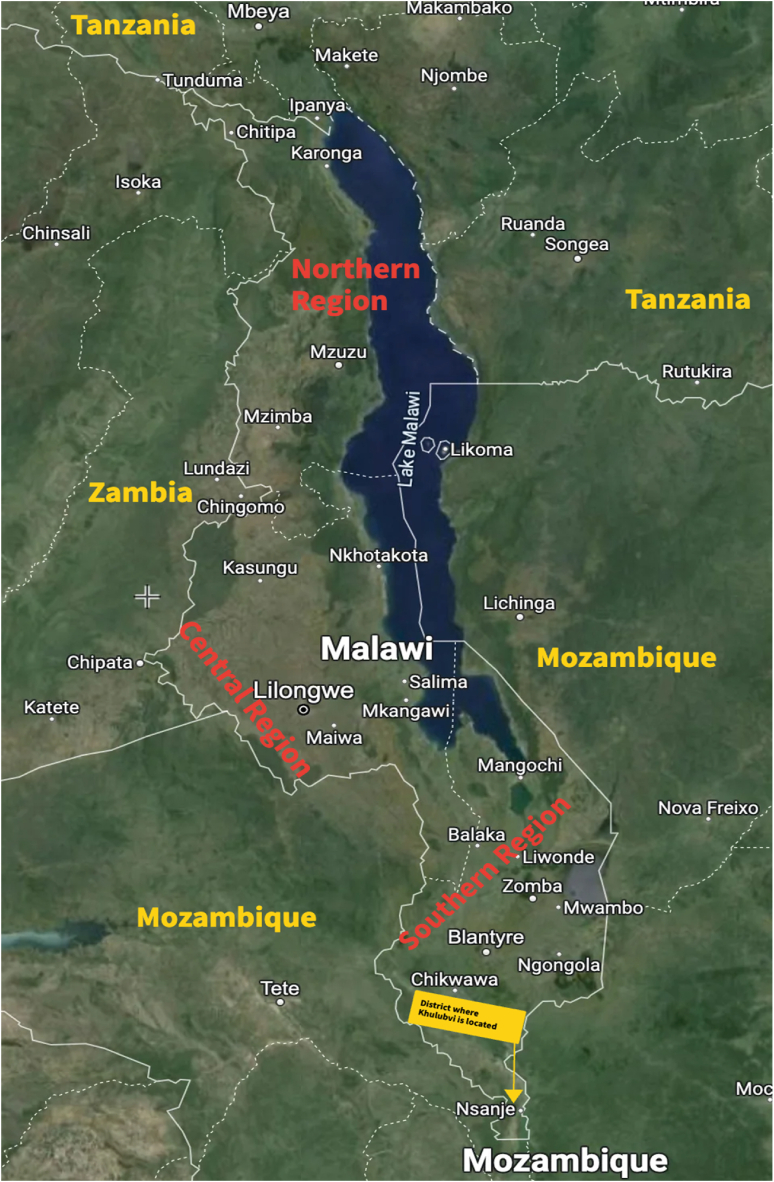
Map showing the location of Malawi and the position of Nsanje District in the Southern Region.

The focal point of this research is the Khulubvi Sacred Shrine, an esteemed heritage site located in the Nsanje District in the Southern Region of Malawi. Malawi is a small multiethnic country in Africa. This landlocked nation is situated in Southeastern Africa [55], geographically delineated by its latitudinal extents ranging from approximately 17.1295° South to 9.3683° South, while its longitude spans from approximately 32.6709° East to 35.9186° East. These coordinates enclose Malawi's varied topographies, extending from the eastern shores of Lake Malawi to its western frontiers, with Mozambique to the east and South West, Zambia to the West and North West, and the United Republic of Tanzania to the North and North East [56]. Fig. 1 is the map illustrating the position of Malawi to its neighboring countries and the region in which Nsanje District, which hosts the Khulubvi Sacred Rain Shrine, is located.

**Fig. 2:**
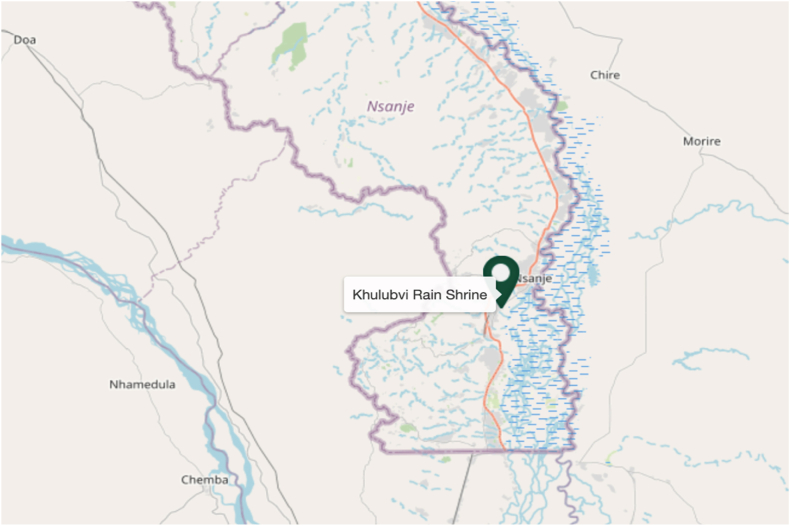
Map showing the position of Khulubvi Sacred Rain Shrine in Nsanje.

The shrine is at the coordinates 16°55′0″ South and 35°00′0″ East. As documented by UNESCO, this location identifies the precise geographical positioning of the shrine within Malawi. In Fig. 2, the Khulubvi Sacred Rain Shrine is meticulously charted in the southern region of Malawi, as indicated by the pin labeled "Khulubvi Rain Shrine."

Nestled near Nsanje [25], this culturally significant site lies along the M1 road, a major arterial route connecting various parts of the country. The map provides a detailed depiction of the area's geographical context, illustrating the network of minor roads, the diverse landscape, and the proximity to other notable landmarks and urban centers.

**Fig. 3:**
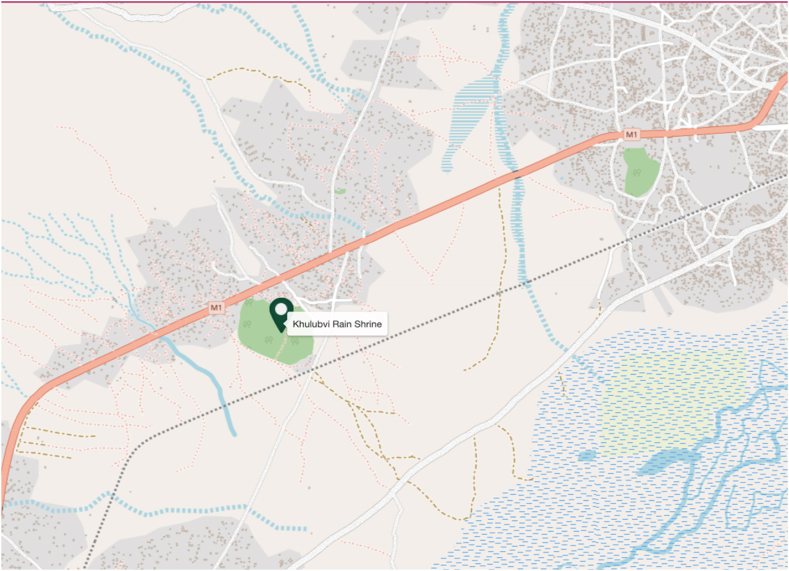
A partial map of Nsanje illustrating the shrine's accessibility. M1 is a tarmacall-weatherr road.

The cartographic representation in Fig. 3 underscores the shrine's accessibility and significance to the surrounding settlements and natural features.

## 5 Materials and methods

This qualitative research approach employs an exploratory research design, focusing on in-depth interviews and thematic analysis to explore the role of community participation in preserving and conserving the Khulubvi Traditional Temple. While capitalizing on the qualitative methods for a deep understanding of participants' perspectives and capturing the richness and complexity of CH preservation, the exploratory design helps understand the topic in-depth and identify potential patterns and themes. Data was collected between 23 July and December 15, 2022.

Purposive sampling was used to select 21 participants [based on data saturation] with direct involvement or knowledge of the Khulubvi Traditional Temple. With this sampling strategy, we envisaged several methodological challenges mitigated through rigorous procedures. The inherent subjectivity of the sampling process was addressed by establishing transparent, well-defined criteria for participant selection, ensuring a systematic approach.

**Fig. 4:**
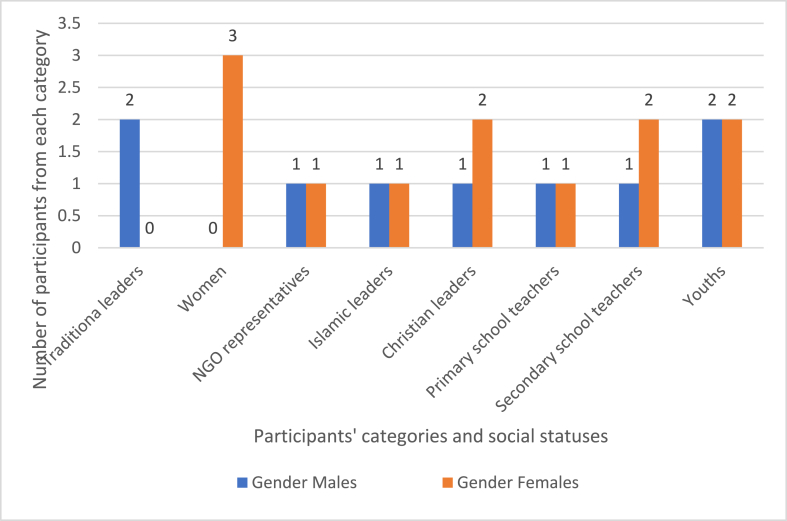
Participants' characteristics.

To counter the limitation of generalizability, we employed data triangulation, incorporating various data sources to substantiate our findings. This includes community members, traditional leaders, representatives from Non-Governmental Organizations, religious institutions, and other stakeholders. Sample demographics are illustrated in Fig. 4.

Reflexivity played a crucial role as we continuously reflected on and acknowledged potential biases, thereby enhancing the credibility of our research. Involving a peer review process added a layer of scrutiny, fostering a more balanced and unbiased selection of participants. Furthermore, to ensure the reproducibility and transparency of our study, detailed reporting of the sampling process and context was prioritized. Lastly, a concerted effort was made to ensure sample diversity within the constraints of purposive sampling, thereby capturing a broad spectrum of perspectives and experiences. These methodological refinements significantly bolstered the robustness and reliability of our research outcomes.

Guided by the Community-Based Participatory Research (CBPR) framework, it emphasizes active community involvement, collaborative decision-making, and sustainable solutions [CBPR guides the research process, ensuring that the community's voice is central to exploring CH preservation strategies] [54]. Semi-structured, in-depth interviews were conducted with the selected participants.

Informed consent was obtained, emphasizing confidentiality and the voluntary nature of participation. Each session was audio-recorded, subject to participant consent, and later transcribed verbatim for analysis. The transcriptions were then analyzed using thematic analysis.

Participants were encouraged to share their experiences, perceptions, and insights regarding the Khulubvi Traditional Temple. They exhibited a wide array of demographic characteristics. Participant reticence, a common issue in qualitative research, was mitigated by establishing a rapport with participants, reassuring them of confidentiality, and employing empathetic listening.

The interviewers were trained in neutral questioning techniques and self-reflection to minimize their influence on participants' responses to avoid bias. Furthermore, to address the potential for data saturation, we carefully monitored the information gathered and adjusted the number of interviews accordingly. Finally, the challenge of interpreting responses was addressed through a systematic thematic analysis involving multiple researchers to ensure a balanced interpretation of the data.

The participants, spanning various age groups, genders, and social and intellectual backgrounds, offered diverse insights and allowed for a comprehensive exploration of perspectives, ranging from youthful enthusiasm to the wisdom of experience.

The participants exhibited varying levels of education, enriching the conversation with diverse insights, ranging from illiterates to graduates. Besides, regarding occupational diversity, the respondents represented different occupations, including teachers, traditional leaders, and community members engaged in environmental conservation. This occupational diversity brought forth multifaceted perspectives on the challenges faced in preserving the Khulubvi Traditional Temple.

Concerning their cultural and religious insights, the interviewees provided valuable cultural and spiritual insights about the Shrine.

All respondents were community members residing near the Khulubvi Traditional Temple. Their connection to the site infused the discussion with a sense of personal attachment and responsibility. They highlighted the community-driven initiatives, such as the wildlife club, reflecting the proactive approach taken by residents to ensure the Shrine's sustainability.

The interviews conducted in Chisena posed a unique challenge in transcription and translation to English, ensuring the integrity of the data. The transcription process began with native Chisena speakers transcribing the audio recordings verbatim in the original language. This step was crucial to capture the nuances and context of the spoken words. Following this, professional translators, proficient in Chisena and English, translated the transcripts into English.

Moreover, to assure accuracy, a back-translation method was employed where a separate team of bilingual experts back-translated a sample of the English transcripts into Chisena. This process allowed for cross-verification of the translations, ensuring that the essence and meaning of the participants' responses were preserved. Member checking was employed to ensure data validity and reliability. Participants had the opportunity to review the transcripts or summaries of their interviews, providing feedback and clarifications.

Meanwhile, those who could not read were helped to listen to their voices, recorded with their consent. Throughout this process, the original audio recordings served as a reference point to resolve any ambiguities or discrepancies in translation, thereby maintaining the integrity of the data. This approach to transcription and translation was integral to our commitment to methodological rigor and cultural sensitivity in cross-linguistic research.

All data was anonymized, and pseudonyms were used to report the findings to ensure the participants' privacy and anonymity.

Thematic analysis was employed to identify patterns, themes, and categories within the qualitative data. The interview transcripts were coded, and the codes were organized into themes related to CH preservation, community engagement, stakeholder roles, challenges, and solutions.

**Fig. 5:**
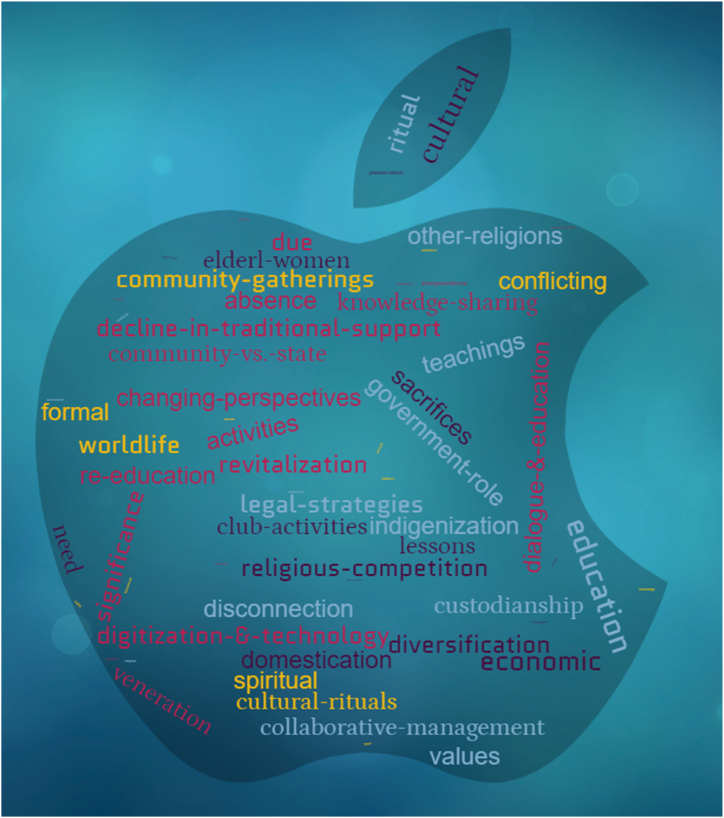
A word cloud of coding prior to theme development.

We developed codes prior to identifying overarching themes in our thematic analysis, a critical step that ensured a systematic and structured approach to examining qualitative data, such as interview transcripts. Fig. 5 illustrates the codes developed using artificial intelligence from WordCloud.com tools.

**Fig. 6:**
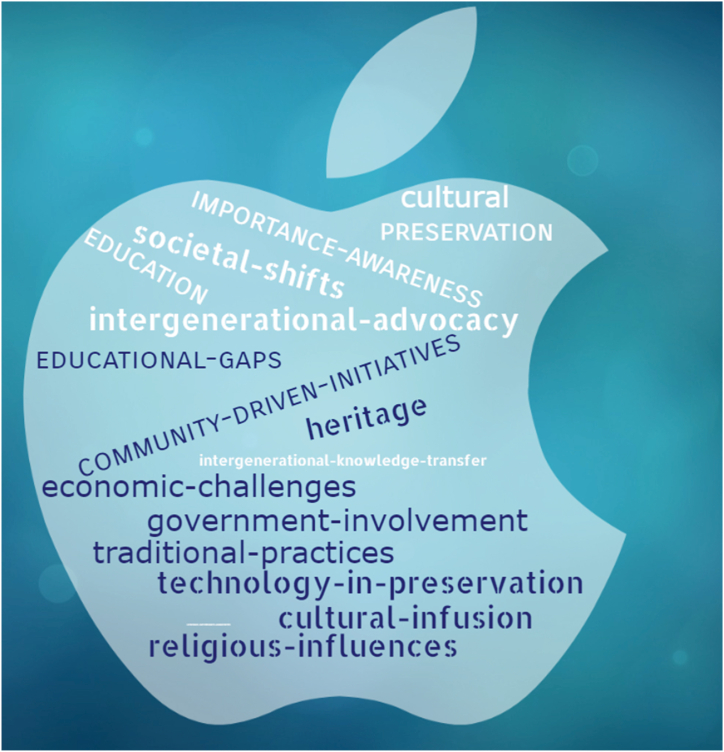
A word cloud of themes.

Consequently, codes acted as foundational elements from which we constructed broader themes, a methodical approach that prevented premature data clustering into themes while reducing the risk of bias and ensuring that all relevant data points were considered. Documenting the progression from coding to theme development provided a clear trace of how our conclusions were drawn, which was essential for the traceability and accountability of the study. Fig. 6 is a word cloud of themes automatically generated from the codes in Fig. 5.

Table 1 maps the research questions against the codes and their corresponding themes to enhance understanding of the relationship between the codes and themes.

**Table 1:** Mapping research questions with codes and themes.

Question	Codes	Themes
1	Wildlife club activities, Community gatherings, Veneration and sacrifices, Spiritual significance, Cultural rituals, Elderly women.	Community driven initiatives
		Traditional practices
		Intergenerational advocacy.
2	Government role, Community vs. State, Collaborative management, Digitization & and technology, Custodianship, Dialogue & and education, and Knowledge sharing.	Government involvement
		Technology in preservation
		Intergenerational knowledge transfer.
3	Other religions, Religious competition, Decline in traditional support, Changing perspectives, Cultural disconnection, Conflicting values due to economic activities.	Religious influences
		Societal shifts
		Economic challenges to cultural preservation.
4	Re-education, Absence of teachings in formal education, Need for indigenization, Ritual revitalization, Lessons diversification and domestication	Educational gaps
		Cultural infusion in education
		Awareness of the importance of heritage in education.

Sentiment mining was also conducted using natural language processing techniques to determine the emotional tone of the interview responses. The participants' positive, neutral, and negative sentiments were analyzed to understand their dynamic perspectives.

## 6 Results

It should be noted that some responses from the participants, though closely involved in the leadership and activities of the shrine, could not differentiate between Mozambique and Congo when describing the origin of the Mang'anja, Lundu, and Mbona. Much as oral history loses details with time, some elements are fundamental and need to be kept intact. They kept mixing the names of countries, indicating the need to preserve and conserve the legacy.

Furthermore, a leader mentioned that every time a significant sacrifice was to be made, all the traditional leaders were called upon. According to custom, it was pretty customary to acknowledge everyone in attendance with "vulgar or abusive words." In his words he said: "*Mfumu iliyonse ikayitanidwa kuti ngana ulipo?*" “Ikati ‘eya ndilipo’, *kenako mfumuyo amayitukwana kuti XXXX.*” Under such circumstances, XXX is a code for offensive or “nasty” language that even the interviewees lacked the courage to say to us.

### 6.1 Thematic analysis

Question 1 What specific CH preservation activities are undertaken by residents to ensure the sustainability and promotion of the Khulubvi Traditional Temple in Malawi?

Exploring community-led preservation activities revealed three fundamental themes: Community-driven initiatives, traditional practices, and intergenerational advocacy. Participants emphasized the proactive role of community-driven initiatives, such as wildlife clubs dedicated to environmental conservation and tree planting, aligning seamlessly with preserving Khulubvi as a forest sanctuary (Mbewe). Traditional practices, including the guidance of traditional leaders and older women in maintaining cultural values, emerged as critical elements (Kabichi). Intergenerational advocacy became evident through teachings, ensuring younger generations understand the cultural significance of the Shrine (Phiri). The following quotations substantiate this analysis.

#### 6.1.1 Theme 1: Community-driven initiatives

We have a wildlife club in the community; we usually meet on weekends. We discuss environmental issues such as climate change and how we can conserve our environment. We discuss soil erosion, droughts, etc., and we emphasize these by addressing deforestation issues. We take the initiative to plant trees in the area so that our place is not bare or dry. I think this club contributes to protecting Khulubvi because Khulubvi is a forest, and we ensure that no one unnecessarily cuts trees on the site—Mbewe, aged 30.

The people entrusted with the authority over the sacred forests and the river associated with the Cult are provided with necessities in terms of food so that they dedicate their time to the protection of the treasure. The government has played the role of food supplier in liaison with the host community—A secondary school teacher.

The Shrine is profoundly integral to our community, steeped in the heritage of the Mbona cult. First, we have the Khulubvi Forest, known as the hair of Mbona, symbolizing the life and protection Mbona offers us. The Ndione waters are the blood of Mbona, representing his sacrifices for the community and the sustenance he provides. The community has people who ensure that the shrine, along with two other huts, replicas of Mbona's house, his wife's, and his wife's maid's dwellings, are maintained regularly. These encapsulate the domestic and spiritual aspects of Mbona's life and teachings. These elements are not just symbols but the living embodiment of our faith and history—Traditional leaders.

Maintaining authenticity is paramount, especially as our practices gain wider exposure. We're committed to educating local and international audiences about the deep spiritual meanings behind our rituals. We host cultural seminars and develop materials that explain the traditions in depth. This educational approach ensures that the practices are not merely viewed as entertainment but respected for their cultural significance and spiritual value.

We understand cultural practices must evolve to stay relevant, especially to our youth. We allow for the organic growth of these traditions, integrating contemporary elements that resonate with younger generations without compromising the core spiritual elements. For example, while we might adapt the form of traditional music or dance, the occasions on which they are performed and the rituals themselves remain unchanged—Traditional leader.

#### 6.1.2 Theme 2: traditional practices

Traditional leaders advocate for cultural values and traditionally revered moral standards. They teach virgins and those reaching puberty how to remain "undefiled." Boys are also initiated into being responsible members of the society—First woman.

The cult's rituals are integrated into the regional politics and the community's social structure, playing a role in unifying the community under common spiritual and cultural concerns. The rituals also reflect and influence the political dynamics between different factions.

#### 6.1.3 Theme 3: intergenerational advocacy

They advocate for decent dressing within the modern setting. For example, older women always advise young girls on the importance of a responsible lifestyle. They tell them to be home in good time and not stay outdoors at night—(Kabichi, 22)

Question 2 How do various stakeholders, including parties, NGOs, religious institutions, and village representatives (VRs), operate to safeguard CH, mainly focusing on the Khulubvi Traditional Temple?

Three pivotal strategies emerged: government involvement, technological integration, and intergenerational knowledge transmission. The significance of government participation, particularly in developing comprehensive educational curricula incorporating traditional heritage, was underscored as a vital element (Jasmine).

Meanwhile, technological advancements, such as digitization and social media platforms, were recognized as practical tools to engage younger demographics, albeit with considerations for existing cultural taboos (Azure). Furthermore, the critical role of intergenerational knowledge transfer, exemplified by custodians imparting cultural wisdom to local communities, was identified as fundamental in maintaining the cultural essence of the Shrine (A Villager). Specific quotations are provided to elucidate these themes.

#### 6.1.4 Theme 4: government involvement

Every society is part of the state. The government has a role to play in preserving and conserving CH. Unfortunately, policy developers, curriculum specialists, and traditional leaders do not come together to develop a holistic curriculum that not only helps society develop scientifically and economically in the wake of the developing technological sphere but also uses the same opportunities availed by technology and innovation to teach traditional heritage of each society. (Jasmine)

From our perspective, the Khulubvi Forest transcends the usual classifications of natural resources. While forests are generally considered state properties to be managed for the national good, Khulubvi is unique. This forest is a sacred grove that grew from Mbona himself, making it a spiritual heritage rather than just a natural resource. Consequently, the Mang'anja people, direct descendants of Mbona and the original custodians of his legacy feel a profound responsibility to protect and manage this forest. We believe it belongs not to the state but to the Mang'anja community, which is its legitimate caretaker- second woman- in a spiritual and cultural sense.

Conflicts over land and resource management are challenging, especially when state interests clash with traditional beliefs. We strive to engage in dialogue with governmental authorities to explain the unique nature of the Khulubvi Forest. The government has always been helpful. Whenever we have great functions, local government officials are made aware. We advocate for a management approach that respects traditional beliefs while aligning with national interests, ideally through a collaborative framework that allows the state and the Mang'anja leaders to oversee its preservation–A male villager.

#### 6.1.5 Theme 5: technology in preservation

Through the ministry responsible for CH, the ministry of education, and the ministry responsible for information (archives), leadership should streamline such heritage into formal programs from primary education. Taking advantage of the new technologies, cloud computing, the digital evolution, and the heightened desire among the youth to use mobile technology, there is a need to digitize such sacred artifacts, CH, and values and infiltrate the social platforms with such knowledge targeting the youths. Keeping cultural richness in its natural form alone faces accentuated threats from lack of self-value among the youths–NGO representative.

I have always been cautious about digitizing or allowing foreigners into the shrine. They may violate some of the code while visiting. Besides, we would not know how they might use the information they gather from here. So we must play safe when adopting some of the preservation methods –Traditionalist.

On this point, one of the individuals closely associated with the Cult had a contrary view:

Digitizing the story of Khuluvi Forest, the assassination of Mbona, and turning his blood into Ndione River would dilute the sacred myths associated with the legend. (A servant of Mbona). Someone can never bring a camera into the sacred Shrine or any related place. So, it is a severe taboo and an abomination to Mbona for putting any of his practices on camera.

#### 6.1.6 Theme 6: intergenerational knowledge transfer

Generally, CH's custody is associated with older people, especially traditional leaders. This makes much sense, primarily since some mysteries may not be easily understood by individuals who are not entirely associated with inner-circle practices. However, suppose we are to keep such heritage symbols safe and for future generations. In that case, such custodians must share information with as many villagers as possible so that society will not lose anything relevant in their absence. (A villager)

The Shrine is sacred. However, its value is fast waning away among contemporary Malawian youths. This is attributable to the inability to mainstream traditional values into the formal curriculum. Formal education adversely impacts the preservation and conservation of our heritage, contrary to what its role should be in ethnocentric education. We have a psychotic addiction to colonial hegemony and the definition of development (Chimango).

Question 3 What are the distinct challenges in preserving the Khulubvi Traditional Temple?

The preservation of the Shrine faced three main challenges: religious, societal, and economic factors. The presence of differing religious doctrines, particularly those conflicting with the Shrine's traditional values, was highlighted as a significant barrier (A Traditionalist). Additionally, societal changes, particularly evolving norms and attitudes toward traditional taboos, posed challenges in maintaining the Shrine's cultural significance (Merissa). Economic factors, such as livelihood encroachment and conflicting activities like commercial sex, undermined the Shrine's sanctity and cultural integrity (Kakholo). Specific participant statements further illustrate these themes.

#### 6.1.7 Theme 7: religious influences

Religious leaders other than those not associated with African Traditional Religion have contributed significantly to the undermining and loss of the values of the Shrine. Some have built their prayer houses whose doctrines counter those of Mbona. (A traditionalist)

There has been a noticeable decline in the cult's patronage, which is concerning. Traditionally, leaders and community members fervently supported the shrine, recognizing its spiritual power and guidance. However, in recent times, some traditional leaders have drifted away. They seem less passionate about the cult, which is reflected in the overall spiritual engagement of our community with the shrine–traditional leader 2.

Several factors contribute to this trend. Primarily, the infiltration of other religions like Islam and Christianity introduced competing beliefs. These religions have gradually swayed some of our leaders and community members, drawing them away from the practices and beliefs that have been our foundation for centuries–Traditional leader.

The absence of a Mulozi presents significant challenges in upholding the traditions and requirements of the cult. Identifying visitors who have adhered to the three-day abstinence rule is crucial for certain ceremonies, as it reflects their respect and readiness to engage with the spiritual practices. Without a Mulozi to perform divinations and discern these individuals, we face difficulties in ensuring that the rituals are conducted according to the prescribed guidelines. This highlights the importance of traditional roles and knowledge holders in maintaining the integrity of our cultural practices.

#### 6.1.8 Theme 8: societal shifts

The other challenge is that the coming in of other religions has intervened too much. The traditional beliefs are usually not in tandem with human rights, lifestyles, and exposure to mainstream media. The sacred Shrine kept sexuality issues a taboo among minors. Nevertheless, today, parents and traditional icons of authority cannot convince their children or subjects on many topics that align with the Khuruvi Sacred Shrine. (Merissa, an ATR advocate)

It is a matter of perceived immediate benefits. In the past, Mbona was revered as a protector who shielded the community from natural calamities like floods and droughts. Nowadays, we face these challenges, yet the relief once attributed to Mbona's intervention is less apparent. Some interpret this as a decline in Mbona's power. However, the truth is more about our community having strayed from his teachings, angering him, which has led to a perceived withdrawal of his protection–One youth.

You know, medical services in the past were not as accessible as today. As a result, people sought solutions for healing at the shrine. As for me, I don't understand how you can convince me that I need Mbona to heal from Malaria when I know that I can get a guaranteed solution from the hospital.

#### 6.1.9 Theme 9: economic challenges to cultural preservation

Much as there is a practice of seeking permission before visiting the Shrine, associated vegetative areas have suffered human encroachment. Some irresponsible villagers around the forest make them creep into the woods in search of trees to earn a living. Some burn charcoal, while others fell trees for poles for timber. (Traditional leader)

Due to land scarcity, some of the land associated with Mbona has been reclaimed for farming. This is a threat to the traditions and sacredness of the Mbona. (Mbona worshiper)

Our youths now have a perverted perspective about traditions and customs. They feel that harnessing such societal values and heritage is being archaic, backward, and uneducated, not realizing that among the functions of a good education lies the ability for self-awareness while becoming a global citizen. (a female villager)

Some economic activities in the community run counter to the values of the Shrine. For example, beer halls and commercial sex activities contradict the values of Mbona, which advocates for abstinence outside marriage. However, the legal framework protects individuals who indulge in commercial sex in Malawi. (Bonondo, a farmer)

Question 4 What pragmatic solutions can be proposed to enhance its preservation and conservation, considering both tangible and intangible aspects of CH?

Addressing educational challenges and solutions led to identifying gaps in formal education, the need for cultural infusion, and raising awareness of the importance of heritage. Participants emphasized the absence of formal teachings about the Shrine, calling for incorporating local history and CH into the curriculum (Gangántha).

Cultural infusion in education was proposed, advocating for incorporating indigenous knowledge and traditions, creating a harmonious blend with Western education (Azure). Raising awareness of the importance of heritage underscored the need for self-realization and embracing local values, ensuring that education does not erode cultural roots (Juliyana). The following quotations highlight the findings.

#### 6.1.10 Theme 10: educational gaps

In my nine years of teaching experience, I have not encountered any subject teachings about the Khulubvi Rain Shrine, yet this defines us as Malawians because our roots are there before we are divided (Gangántha).

What I know about the place is that there was once an animal that lived in the forest, and people used to worship it when they faced calamities, for example, when there was drought or when one was sick. After making sacrifices, all problems were solved (Juliyana).

The community needs reconnection, starting with re-education and returning to its roots. They must remind their people—especially the leaders—of the significance of their traditions and the spiritual guidance Mbona provides. It involves organizing more community gatherings at the shrine, revitalizing the rituals that celebrate and reaffirm their faith, and openly discussing the values Mbona taught them. It's about creating an environment where old and young can learn about the power of Mbona and see its relevance in their lives today. This is not just about preserving a tradition; it is about living their truth–NGO representative.

#### 6.1.11 Theme 11: cultural infusion in education

We need to indigenize knowledge and diversify our sources instead of incorporating what looks Western into the school curriculum. We must re-infuse local historical, religious, and CH into formal learning institutions. We need to understand the difference between westernization and development. Such elements must be infused into the curriculum from the basic education level. (Azure)

#### 6.1.12 Theme 12: awareness of heritage importance in education

We need to domesticate our lessons from multiple sources elsewhere. We need to realize our self-worth and self-belief. We must make our youths understand that education does not mean denying and denouncing one's roots. Instead, it is a meaningful blend of larger societal common values with local flavors and domestic characteristics. (Jasmine)

Preserving our CH while preserving and conserving its secrecy presents a delicate balance. We've adopted modern preservation methods cautiously, ensuring they do not compromise the confidentiality of our rituals and knowledge. While digitization allows for broader dissemination of information, certain aspects of our practices remain inaccessible to the public. For instance, photography is prohibited in specific areas of the shrine, preserving the sacredness and privacy of our ceremonies.

One government official reiterated the sentiments of an NGO's suggestion. In separate interviews, the two alluded to the need to involve the locals when planning commercialization concerning Khulubvi. They suggested a need to develop the area for cultural tourism, as is the case with Chongoni.

Exploring the potential to share the unique cultural and historical significance of the Khulubvi rain shrine with a broader audience could greatly benefit the local community economically. However, we must collaborate closely with the local residents. Their insights and blessings are crucial as we consider ways to responsibly develop and promote the shrine as a sustainable cultural tourism destination– NGO Member Statement

As we contemplate the commercial development of the Khulubvi rain shrine, we must engage with the local community from the outset. Their deep connection to the shrine must guide our efforts. By working together, we can ensure that any development respects and preserves the shrine's cultural integrity and brings tangible benefits to the community.

### 6.2 Sentiment analysis

The sentiment mining analysis of the study reveals a nuanced perspective on preserving the Khulubvi Traditional Temple in Malawi. Positive sentiments are evident in the community's active engagement, with initiatives like wildlife clubs and tree planting reflecting a proactive approach. Traditional practices, guided by leaders and older community members, are viewed positively, emphasizing the importance of CH transmission.

Government involvement is recognized as crucial, although concerns exist about the need for a comprehensive curriculum that integrates traditional values. Additionally, participants acknowledge the potential of technology, particularly digitization and social media, in preserving CH despite challenges related to conventional taboos.

However, negative sentiments emerge concerning the diminishing value of the Shrine among the youth due to modern influences and formal education. Challenges include economic activities and encroachment, posing threats to cultural preservation. Resistance to digitization is noted, particularly among individuals closely associated with the Shrine, highlighting the complexities of balancing tradition and modernity in heritage preservation efforts. We provide a detailed discussion of the sentiment analysis in the following paragraphs:

The sentiment analysis of the in-depth semi-structured interviews regarding preserving the Khulubvi Traditional Temple in Malawi reveals the participants' complex tapestry of emotions and perspectives. The exploration of community-led preservation activities uncovers a sense of proactive engagement and commitment among the residents. Participants like Mbewe, aged 30, are deeply dedicated to environmental conservation and tree planting, fostering a sense of responsibility towards the Shrine. This sentiment is further amplified by the traditional practices advocated by leaders, who emphasize the importance of cultural values and moral standards, fostering a sentiment of reverence and respect.

In addition, intergenerational advocacy emerges as a powerful sentiment, underscoring the commitment to passing down cultural knowledge to younger generations. Phiri's statement, highlighting the absence of formal teachings but expressing the importance of sacrifices made in the past, evokes a sense of cultural resilience and continuity despite the challenges.

Furthermore, the sentiment around government involvement combines hope and frustration. Participants like Jasmine express optimism about the role of the government, emphasizing the need for holistic curriculum development. However, there is a note of disillusionment as well, with concerns raised about the lack of collaboration and holistic approaches, reflecting a sentiment of missed opportunities and unfulfilled potential.

On the contrary, the theme of technology in preservation elicits a sentiment of cautious optimism. Azure's perspective on digitization and social media outreach reflects hope in leveraging modern tools to engage the youth effectively. However, the view is nuanced, with concerns raised by a servant of Mbona about the potential dilution of sacred myths associated with the Shrine due to digitization, highlighting a feeling of protective traditionalism.

On a different note, the sentiment around intergenerational knowledge transfer is imbued with a sense of urgency and responsibility. The emphasis on sharing information with as many villagers as possible, as articulated by a villager, reflects a sentiment of communal guardianship and collective ownership. Chimango's statement, expressing concern about the erosion of heritage due to modern education, evokes an emotion of lamentation and urgency, underscoring the need for immediate action.

Moreover, the challenges in preserving the Shrine evoke concern, frustration, and urgency. The sentiment around religious influences is one of conflict, with traditionalists like A traditionalist expressing frustration over the undermining of shrine values by other religions, reflecting a sentiment of cultural clash and erosion.

Not only that, societal shifts elicit sentiments of bewilderment and helplessness. An African Traditional Religions advocate, Merissa expresses concern about the evolving norms and the challenge of convincing younger generations, reflecting a sentiment of cultural disorientation and generational divide. Economic challenges voiced by participants evoke desperation and powerlessness, echoing a feeling of financial struggle and cultural compromise.

However, the sentiments around pragmatic solutions are characterized by hope, determination, and a call to action. The sentiment of educational gaps is tinged with frustration and disappointment. Phiri and Manase lament the absence of formal teachings about the Shrine, reflecting an idea of missed opportunities and cultural neglect.

Finally, cultural infusion in education evokes a sentiment of empowerment and reclamation. Azure's call to indigenize knowledge and diversify sources reflects cultural pride and self-affirmation, emphasizing the importance of embracing local heritage in education. Jasmine's sentiment underscores the need for self-realization and embracing local values, highlighting a feeling of self-discovery and cultural identity.

In summary, the sentiment analysis of the interviews reflects a complex interplay of emotions, ranging from hope and determination to frustration and concern. Participants express a deep sense of responsibility towards preserving the Khulubvi Traditional Temple, underscored by a commitment to intergenerational knowledge transfer and a call for holistic approaches that integrate traditional heritage into formal education. Despite challenges and conflicts, there is a prevailing sentiment of resilience and cultural pride, emphasizing the importance of preserving and conserving the Shrine for future generations.

## 7 Discussion

### 7.1 Mbona's waning teachings and values

The discourse on Mbona's teachings, which advocate for harmony between humanity and nature, as well as community unity and support, exemplifies the principles of 10.13039/100015252CBPR. The local community's adherence to these values, particularly in response to environmental challenges, showcases the application of traditional wisdom in conjunction with scientific approaches to environmental management. This integration not only sustains the physical environment but also fortifies community resilience, embodying a holistic approach to cultural and environmental preservation that is rooted in participatory practices.

However, some elements of the cultural traditions are gradually lost from the Khulubvi Shrine. These include vocabulary, as seen in the inability of the traditional leader to speak out the abusive words used during roll calls. Consistent with scholarship on religious history, this could be attributed to the possible influence of Christianity, which prohibits vulgar language [32]. However, it also reveals challenges associated with cultural preservation, as some elements succumb to the pressures of cultural globalization. The observed erosion of specific cultural elements, such as traditional vocabulary at the Khulubvi Shrine, underscores the challenges of cultural preservation amidst global influences. Through a CBPR approach, engaging the community in actively documenting and revitalizing these traditions fosters a shared commitment to heritage preservation. This engagement ensures that cultural adaptations to modern influences are managed in ways that reinforce, rather than dilute, cultural identity, enhancing the shrine's relevance across generations.

Banda's account highlights traditional leaders' pivotal role and influence as custodians, preserving the Shrine's sanctity and heritage by guiding community activities and ensuring adherence to cultural practices, making them key figures in educating the community about the Shrine. Paradoxically, some traditional leaders are unable to articulate a clear narrative of what happened in Mozambique as opposed to Congo. This is in line with Wrigley's argument that some heritage is distorted due to lack of proper records [12] in a comment on Schoffeleers'works [16]. Similarly, there are variations in the narratives of different respondents. This diversity of versions indicates a lack of dependable record of the myths about Mbona, consistent with Wrigley's arguments [12]. Besides, the variations corroborate extant claims that the three streams of powers in the Mbona cult emphasize different elements of the myths [12,25].

As the cult is rooted in the belief that living a morally acceptable life pleases Mbones, who then protects the community from calamities, teaching the youths a traditionally sanctioned lifestyle subsequently preserves the shrine. These lessons ensure the vivacity of the shrine.

However, the absence of the spirit medium poses a formidable setback: some people may be tempted to indulge in abominable acts. For example, people visiting the shrine may violate core values such as putting on black attire. Some may be tempted to include different colors underneath. Others may not observe abstinence before visiting the shrine. More significantly, it may not be easy to physically check visitors from outside the community as this may violate their privacy.

### 7.2 Lack of formal educational initiatives

Amidst the waning teachings of Mbona, the shrine is confronted with a lack of formal curriculum. Phiri's concern about the absence of shrine-related education in the formal curriculum points to a significant educational gap. This gap hampers community awareness and dilutes cultural identity. Phiri's statement reflects the challenges faced in preserving CH due to a lack of formal education. Studies emphasize the centrality of ritual and religious practices in community identity, similar to how the Khulubvi Temple serves as a focal point for cultural and spiritual activities in Malawi [16,17,22]. This aligns with [37], who suggests that integrating these practices into education systems can bridge the gap between tradition and modernity, ensuring the transfer of cultural values across generations. Addressing this gap is critical to preserving cultural identity and community cohesion, aligning with the first and third study objectives.

Some youths lamented that the formal curriculum does not disseminate content about the cult. On the contrary, scientific evidence highlights the critical role of education in CH preservation, suggesting integrating local history and CH into formal educational curricula to foster a deeper understanding and appreciation among younger generations [31]. Such measures would also enhance co-existence and appreciation of each others’ culture in multi-ethnic states like Malawi. One of the aims of an ethnocentric curriculum is to cultivate cultural awareness. Therefore, the intervention could mitigate the challenges of generational disengagement by making cultural education relevant and accessible to the youth, thereby ensuring the continuity of cultural practices.

However, being a multi-ethnic country daunted by heavily tribalistic and regionalistic politics, developing a formal curriculum across a diversity of CH will be met with pertinent challenges underpinned by philosophical, sociological, economic, and political factors. As such, there is a need to navigate such a landscape with care. Applying CBPR principles and involving community members in the development of educational content ensures that such initiatives are culturally congruent and educationally effective. This collaborative approach not only bridges the gap between traditional knowledge and formal education systems but also strengthens community agency in cultural stewardship.

### 7.3 Environmental conservation and sustainable cultural practices

Despite the absence of formal education about the shrine, the community has registered progress in environmental sustainability. Kurin and Flint explore the relationship between environmental sustainability and CH, which are the core teachings of Mbona, noting the importance of maintaining the ecological integrity of sacred sites [26,57]. Everyone is prohibited from going into sacred places without appropriate approval to preserve the environment, contributing to cosmic stability. The leaders make it a taboo to poach Mbona's children (fauna) or “fell” his hair (flora), but the result remains significant for biodiversity protection and for minimizing greenhouse emissions. Mbewe's interview highlights community-driven environmental conservation efforts.

The wildlife club's activities promote ecological awareness and indirectly contribute to preserving the Shrine. The community ensures its sustainability by protecting the natural habitat around the Shrine. This aligns with the first and third study objectives, underscoring the community's dedicated engagement and proposing viable solutions for heritage preservation. This perspective is crucial in understanding community-driven conservation efforts at Khulubvi, corroborating literature on sustainable management of natural resources is integral to the preservation of cultural sites [11,16,17,25]. Preserving and conserving this site is a matter of environmental concern and a cultural imperative. This dual approach supports the notion that cultural and natural heritage are profoundly interconnected and that effective management strategies must address both dimensions [33]. The community-driven environmental conservation efforts surrounding the Khulubvi Shrine illustrate CBPR in action. Initiatives such as restricting access to sacred spaces and sustainable resource management reflect a community-centric approach to environmental stewardship. These practices, deeply rooted in cultural and spiritual values, highlight the community's proactive role in maintaining the ecological integrity of their sacred sites, thus ensuring the preservation of both cultural and natural heritage.

### 7.4 Socio-economic factors as preservation challenges

Traditional leaders indicated they prevent anyone from looking for resources from the sacred place for personal use. For example, encroachment, poaching, or logging are taken as taboos. Such findings corroborate extant research: Economic activities, such as commercial agriculture and tourism, pose significant challenges to CH preservation by potentially undermining traditional practices and the sanctity of sacred sites. The literature indicates that while economic development can provide resources for preservation, it often introduces new threats that must be managed carefully to avoid disrupting traditional cultural expressions [46].

### 7.5 Successes and challenges of community engagement

The conservation efforts above are achieved thanks to the social fabric enabling people to work as a community. The significance of community engagement in the preservation of CH, as discussed in the literature, underscores the pivotal role communities play in preserving and conserving their cultural practices. For instance, members are involved in contributing resources for sacrifices. Men, women, and the youths are involved in rituals, songs, and cultural practices in the night, through which dissemination of their narrative is done. This involvement is essential, reflecting a collective memory and identity that are intricately woven into the social fabric of the Mang'anja people [14,17,25]. This aligns with the CBPR framework, which emphasizes active community participation in heritage management [52,54].

However, some members are more involved than others. For instance, women in their menopause age [16] are more involved than girls [16]. Boys feel less involved than traditional leaders in decision-making, and some traditional leaders feel less involved in decision-making and in leading ritual ceremonies than others, causing a loss of interest, consistent with the literature [34]. We attribute this variety in patronage, allegiance, and consistency to the impact of the three streams of power MacGaffey talks about [25]. Preservation of CH invokes questions of who benefits most.

Regarding visitors to the site, the custodians do not benefit directly from the income generated when such tourists enter Malawi. All these challenges need a closer look to make more Mang'anja members more proud of their inheritance. Where one stratum finds more sidelined, it is logical that it will withdraw its allegiance. This would be an act against the core values of Mbona– unity and oneness. This discrepancy may make some community sectors feel sidelined and withdraw their limited involvement in communal services, such as not contributing resources for libations.

### 7.6 Resistance to modern preservation methods

The resistance to modern preservation methods, such as digitization, reflects a broader tension between traditional secrecy and the need for contemporary preservation methods. Khulubvi standards do not accept some practices and sites to be on camera. The reasons could stem from different culturally valid reasons. Besides, using artificial intelligence methods to preserve such heritage invokes challenges of ownership, control, accuracy, and motives.

Kurin discusses the potential of technology in enhancing the documentation and preservation of CH [57]. This mirrors the findings where modern methods like digital archiving and virtual reconstructions are suggested to help preserve the tangible and intangible aspects of the Khulubvi Temple, aligning with a plethora of scholarship about advocacy for technology's role in cultural preservation. However, fears that some cult members have about modern preservation methods under the guise of “cultural heritage at risk” are not surprising, as prevalent literature attests that some preservation measures may not benefit the host community [1,29,30]. Besides, the digital divide and information gaps pose a severe threat. Opportunists may exploit such gaps to their advantage. Technological barriers, such as lack of access and digital literacy, hinder the implementation of advanced preservation methods. Through the CBPR lens, this resistance is addressed by engaging the community in dialogues to negotiate acceptable methods of technology integration that respect cultural norms and enhance heritage preservation efforts.

### 7.7 Economic demands in conservation and preservation

Moreover, adequate funding is essential for executing heritage preservation projects effectively. Limited financial resources often result in compromised project quality and scope. Funding challenges necessitate innovative financing models, public-private partnerships, and grant applications to secure resources for sustainable preservation endeavors. Unfortunately, the economic demands of such projects in a low-income state would require donor funding, which usually comes under conditions that may contravene the desires of the community traditions. The literature suggests that while modern technologies offer new ways to document and preserve CH, they must be employed thoughtfully to respect and preserve the integrity of traditional practices [44]. Bridging the digital divide requires capacity-building initiatives, training programs, and partnerships with tech organizations to enhance community access to digital tools [58].

### 7.8 Commercialization of cultural heritage

Suggestions from an NGO representative to commercialize the place raise some concerns. Malawi is a low-income country with a humble infrastructure. Opening up Khuluvi for ecotourism would tremendously lead to facelifting the site [34,35]. However, if poorly designed, such development may lead to an imbalance in the spiritual and cultural ecosystems. Careless ecotourism and cultural tourism influence the available resources, which comprise the environmental resources [26]. Besides, extant studies indicate that such commercialization would bring a milliard of socio-economic complications [26]. These findings are consistent with Emmanuel's lamentation about the misrepresentation of African cults in Nollywood for commercial purposes and corroborate other studies that discuss fears of loss of authenticity when exposed to the outside world [26,45,47]. The commercial pressures facing the Khulubvi Temple, such as tourism and agriculture, require careful management to prevent cultural dilution, a concern Foster and Gilman also highlighted in their work on economic impacts on cultural practices [37]. The exploration of socio-economic factors reveals the intricate relationship between economic development and cultural preservation. The CBPR framework facilitates a community-engaged approach to navigating these challenges, ensuring that economic initiatives such as tourism and commercial development are aligned with the community's cultural preservation goals. This collaborative strategy fosters economic practices that support, rather than undermine, the sanctity and sustainability of the 10.13039/100003698CH.

### 7.9 Challenges of modernization and globalization

Modernization, on the one hand, can boost CH's recognition on the international scene. Much as the shrine has been proposed to be recognized by UNESCO to boost global support [11], there is a need to build international partnerships, enhance online presence, and participate in global cultural events. This integrated approach aims to increase visibility, attract resources, and foster worldwide appreciation for Khulubvi's CH.

On the other hand, the negative impacts of globalization and modernization on Khulubvi include cultural homogenization, loss of traditional skills, and environmental degradation, all of which threaten the preservation of its unique CH. For example, the findings that some youths withdraw their patronage from the cult due to alternative solutions corroborate theories that emphasize that people will withdraw their interests from what is not beneficial. Contrary to what was common in the pre-colonial period, some youths prefer medical solutions from hospitals to believing in Mbona's healing power. This is an impact of modernization, cultural globalization, and technological advancement [[38], [39], [40]]. Noyes and McGregor address the impacts of modernization and globalization on traditional cultures, particularly how economic developments can threaten CH sites [59]. When cultures are too rigid, they face the challenge of existence. If the youths are accorded reasonable space to accommodate new aspects of culture with a careful balance, such cultures thrive [35].

### 7.10 Technological innovations in preservation

Communities often resist adopting new preservation methods that challenge traditional practices and beliefs. This resistance arises due to the deeply rooted cultural norms and historical significance of existing heritage practices.

Besides, in line with experiences around the Chongoni heritage site, where locals do not benefit enough from commercializing the site, this resistance reflects deep fears about how the locals would benefit from such measures, both culturally and economically [34]. Overcoming this resistance demands culturally sensitive approaches and collaborative efforts that respect and integrate local traditions into preservation initiatives. This aligns with Johnson's view: Ethical considerations, including cultural respect and representation, are paramount in preservation efforts. Missteps in cultural expression can lead to tension and conflict within the community. Ethical guidelines, cultural sensitivity training, and community consultations are essential in addressing these concerns.

The findings comprehensively understand the challenges and opportunities in preserving the Khulubvi Traditional Temple. The sentiment analysis revealed a mix of emotions expressed by the interviewees. The positive sentiment reflected confidence in traditional leaders' roles, while the neutral tone provided factual information. On the other hand, the opposing view highlighted disappointment regarding the lack of educational focus on the Shrine, and Mbewe's positive sentiment highlighted community enthusiasm and engagement in conservation efforts.

### 7.11 Legal and policy frameworks

Manase's narrative illuminates the historical and religious significance of the Shrine. The account of ancient rituals and sacrifices during calamities emphasizes the Shrine's deep-rooted cultural value. This historical context is crucial for understanding the Shrine's role in the community and its relevance in the contemporary context. It aligns with the second study objective, examining stakeholder operations and highlighting the religious institutions' historical significance and contribution to the Shrine's preservation. Blake discusses the complexities of defining and protecting CH under international law; a challenge echoed in the findings where legal ambiguities complicate the preservation of the Khulubvi Temple [3].

Therefore, any "external stakeholders," such as the Government, must tread circumspectly to avoid clashes with the community. Balancing CH preservation with community development initiatives poses complex challenges. Conflicts often arise between those advocating for heritage conservation or preservation and developers seeking infrastructural progress. Integrating heritage impact assessments into development planning and fostering stakeholder dialogue can mitigate conflicts [11]. Therefore, consistent with local studies, there is a need for policies that are culturally sensitive and inclusive of local community needs, which would support the legal recognition and protection of the Temple [34].

Challenges related to navigating and complying with legal frameworks are a recurring theme in the literature. It is suggested that creating policies that recognize and protect the intangible aspects of CH is crucial. This requires a balance between respecting traditional practices and meeting modern legal standards, thereby ensuring that CH management aligns with both local values and national interests [30].

## 8 Conclusion

This study highlights significant challenges and opportunities in preserving the Khulubvi Traditional Temple through active community participation. While the research successfully illuminates the roles of community engagement, stakeholder operations, and technological integration, it also reveals key shortcomings that must be addressed. One notable shortcoming is the lack of formal educational initiatives that incorporate traditional heritage into the curriculum. This gap hampers awareness and engagement, particularly among younger generations, leading to a dilution of cultural identity and community cohesion. Additionally, the resistance to modern preservation methods, such as digitization, underscores the tension between maintaining traditional secrecy and embracing contemporary preservation techniques. This resistance, rooted in cultural norms, poses a challenge to effectively documenting and safeguarding the shrine's heritage.

### 8.1 Limitations of the study

Despite the valuable insights provided, the study has several limitations. Firstly, the reliance on qualitative data may introduce biases, as the findings are based on subjective interpretations of interviews and focus groups. Additionally, the study did not account for potential external influences, such as political or economic changes, that might affect community engagement and preservation efforts. These limitations suggest a need for more comprehensive and diverse methodological approaches in future studies.

### 8.2 Recommendations for future research

Future research should focus on developing culturally sensitive educational programs that integrate traditional heritage into formal curricula, fostering greater awareness and engagement among the youth. Additionally, exploring collaborative strategies that balance traditional preservation methods with modern technologies will be crucial. Addressing these gaps will enhance the resilience of cultural practices and ensure the sustainable preservation of the Khulubvi Traditional Temple.

Aligning future studies with the current findings, researchers should investigate the long-term impacts of educational initiatives on cultural preservation and community cohesion, as well as the effectiveness of integrating modern technologies in a culturally respectful manner. This approach will provide a comprehensive understanding of how to navigate the complexities of cultural heritage preservation in the face of modernization and globalization.

This study illuminates the path forward for preserving CH, emphasizing the significance of community-driven initiatives, education, and thoughtful technology integration. The Khulubvi Traditional Temple stands as a testament to the resilience of cultural traditions, and it is imperative to protect and cherish it for generations to come.

### 8.3 Areas for further study

The study proposes conducting in-depth ethnographic research to delve deeper into the intricate cultural nuances, beliefs, and rituals associated with the Khulubvi Traditional Temple, providing a richer understanding of its significance and challenges.

## Data availability statement

Data for this study is available upon request through the corresponding author.

## Ethical issues

In close accordance with the Declaration of Helsinki of 1975, the study was approved by the lead author's university, and all subjects gave their informed consent for inclusion before participating. Mzuzu University Research Ethics Committee provided the Ethical clearance under the research project with reference number Ref No: MZUNIREC/DOR/21/55; under the project Performance in Malawian Education System, who is the smartest?

The study did not involve any minors. The authors complied with all relevant ethical regulations. The study does not report population-level health estimates that include quantitative data on health indicators at the global, regional, national, or subnational levels. Nor does the study involve human participant data related in any way to human health. Besides, the study does not contain photographs, images, or videos showing humans or parts of humans. The study does not involve experimentation on animals nor includes Cell Lines.

Moreover, the study does not report structures of biological macromolecules derived from/determined using X-ray crystallography, Cryo-EM, or NMR, or new small molecule structure(s), nor use statistical techniques.

This submission acknowledges and adheres to ethical standards stipulated in the journal's author's ethical guidelines concerning the use of generative AI and authorship contribution.

All co-authors saw and approved the final version of the paper and agreed to its submission for publication.

## CRediT authorship contribution statement

**Lazarus Obed Livingstone Banda:** Writing – review & editing, Visualization, Validation, Supervision, Software, Resources, Project administration, Methodology, Investigation, Funding acquisition, Formal analysis, Data curation. **Chigonjetso Victoria Banda:** Validation, Resources, Methodology, Data curation. **Jane Thokozani Banda:** Writing – review & editing, Writing – original draft, Project administration, Methodology, Investigation, Funding acquisition, Formal analysis, Data curation, Conceptualization. **Tapiwa Singini:** Writing – original draft, Software, Resources, Data curation, Conceptualization.

## Declaration of competing interest

The authors declare that they have no known competing financial interests or personal relationships that could have appeared to influence the work reported in this paper.
